# Red and blue wavelengths affect the morphology, energy use efficiency and nutritional content of lettuce (*Lactuca sativa* L.)

**DOI:** 10.1038/s41598-021-87911-7

**Published:** 2021-04-16

**Authors:** Xiao-li Chen, You-li Li, Li-chun Wang, Wen-zhong Guo

**Affiliations:** 1 Beijing Research Center of Intelligent Equipment for Agriculture, Beijing Academy of Agriculture and Forestry Sciences, Beijing, 100097 China; 2 Key Laboratory of Urban Agriculture (North China), Ministry of Agriculture and Rural Affairs, Beijing, China

**Keywords:** Plant sciences, Light responses

## Abstract

Since red (R) and blue (B) LED light has different quantum efficiency and photoelectric conversion efficiency, mixed RB with different proportions of R and B results in varied energy consumption. In order to improve the energy use efficiency of the closed-type plant production systems, the effects of R and B proportions on the electric use efficiency (EUE), light use efficiency (LUE) as well as the quality of butter leaf lettuce were evaluated in this study. Lettuce seedlings were cultivated in a plant factory with artificial lighting (PFAL) and subjected to eleven combinations of R and B (100%R, 90%R, 80%R, 70%R, 60%R, 50%R, 40%R, 30%R, 20%R, 10%R, 0%R; the rest of the photons in each treatment were B) with the same total photosynthetic photon flux density (PPFD) and photoperiod (200 ± 3 μmol·m^−2^·s^−1^, 16 h) for 35 days. The results showed that palpable petiole distortion appeared when R proportion was more than 70% and the distortion was aggravated with the increase of R proportion. The highest EUE and LUE were both detected in lettuce under 90%R treatment, which were respectively 3.64% and 1.20%. The least number of photons and the least electricity amount required to produce 1 g dry weight of lettuce was respectively 2.92 mol and 1.67 MJ, which were both detected in lettuce treated with 90%R. The sucrose content in lettuce treated with more than 50%R was significantly higher than those treated with less than 50%R (50%R included). Lettuce treated with 80%R possessed the highest soluble sugar content as well as the lowest crude fiber and nitrate content (not significantly different with the minimum values). R proportion exceeding 50% in mixed RB light was beneficial to the accumulation of hexose and sucrose, as well as the decomposition of nitrate in lettuce. The vitamin C content in lettuce treated with 100%R was significantly higher than that in lettuce under other treatments in the study. On the whole, the study indicated that the proportions of R and B affected the energy use efficiency and quality of lettuce in closed plant factory, however the responses of plants to the proportions of R and B varied according to different indexes. Thus, some indexes of top priority should be determined before choosing the optimal proportions of R and B.

## Introduction

Since light spectrum strongly affects plant development and physiology, light environment control is an effective mean to improve plant growth and quality in controlled environment agriculture (CEA). Light environment control includes four aspects that are light intensity, light quality, light period and light distribution, among which, light quality acts the most complicated effects on plant physiology. R with a peak wavelength of 660 nm closely matching a peak absorbance of chlorophyll has important function on plant growth and biomass accumulation^1^. It was reported that if only monochromatic light was allowed or available during lettuce cultivation, R might be the only light quality that can basically meet the growth, yield and quality requirements of lettuce^2^. Some reports also proved that plants under monochromatic R showed higher dry weight than those under combinations of R and B^3,4^.

Although R played irreplaceable role during plant cultivation, the mixture of R and B has been reported more beneficial for many vegetables and crops including pepper, cucumber, tomato, wheat and rice. For example, it has been reported that net photosynthetic rate (Pn) and dry weight could be enhanced by combinations of R and B compared with monochromatic R^5–9^. At present, there have been many studies involving the effects of R/B ratio on plants, but it is still not clear whether the positive effects of R and B are quantitative progressive responses or a qualitative responses. Although Chen^10^ reported that plant responses to light quality were specific to species, there has been no final conclusion on the optimal R and B proportions even for the same species. Taking lettuce for example, Wang^11^ testing R/B ratios of 12, 8, 4, 1 and monochromatic R and B in the development of lettuce found that lettuce had higher dry weight with the increase of R/B ratio until 12. However, lettuce growth was not constantly improved with the increase of R proportion in the experiments conducted by Wen^12^ and Zhang^13^ (Wen testing R/B ratios of 10, 8, 6 showed that 8 was the best ratio for lettuce growth; Zhang testing R/B ratios of 9 and 4 found that 4 better effectively improved lettuce growth). Dougher and Bugbee^14^ reported that 2%B in the combinations of R and B was the most effective for lettuce biomass accumulation. Additionally, Son and Oh^15^ testing R/B ratios of 100:0, 87:13, 74:26, 65:35, 53:47 and 41:59 reported that lettuce biomass under monochromatic R were greater than those under mixed RB, which were supported by the study of Wollaeger^3^. Thus, the synergetic or antagonistic effects of R and B on lettuce is still confused, more studies need to be conducted. Moreover, most related studies including these mentioned above set specific ratios of R and B and took R as mainly light^16^, thus it is difficult to compare the results in different studies due to the lack of consistent gradient change for R and B proportion. Equal and symmetrical gradient change for R and B proportion in one study may help to better present the plant responses to the changes of R and B proportions.

The application of light-emitting diodes (LEDs) in horticulture is constantly expanding during these years, mainly due to the advantages of controllable spectral composition, linear photon output, cool emitting surface, and long durability^17,18^. Although the costs of LEDs have been decreasing to facilitate their use in horticultural applications, the energy consumption of LED lighting source is still a restrictive factor for their large-scale applications in CEA. As mainly used light qualities, numerous studies on the irradiation strategy (i.e. light intensity, light period and light quality) of red and blue LEDs in a plant factory have been reported, however the LUE or EUE of plants have rarely been concerned. According to the light emitting principle of LED, photon output is linear with the electrical input current or the actual working power^19^. However, due to the different fabricate technology of LED chips, the linear function between the PPFD and the actual working power is not consistent for red and blue LEDs. In other words, combinations of R and B with the same total PPFD but different proportions of R and B show varied energy consumption since that R and B has different quantum efficiency and photoelectric conversion efficiency. Previous studies focusing on the R/B ratio rarely paid attention to the energy consumption nor the energy use efficiency in different R/B ratio light treatment. In order to improve the energy efficiency of the LEDs in closed-type plant production systems, it is necessary to study the effects of different R and B proportions on not only the quality but also the EUE and LUE of plants.

Thus, the present study aimed at determining the effects of different R and B proportions on lettuce morphology, nutritional content and energy use efficiency. Mixed RB with continuous changes of R and B proportions were conducted and morphology, biomass, pigments, carbohydrate, vitamin C, nitrate as well as EUE and LUE of lettuce were evaluated. The results obtained from the study are expected to supply theoretical basis for the practical use of red and blue LEDs in closed-type plant production systems.

## Methodology

### Experimental design and growth conditions

**Figure 1:**
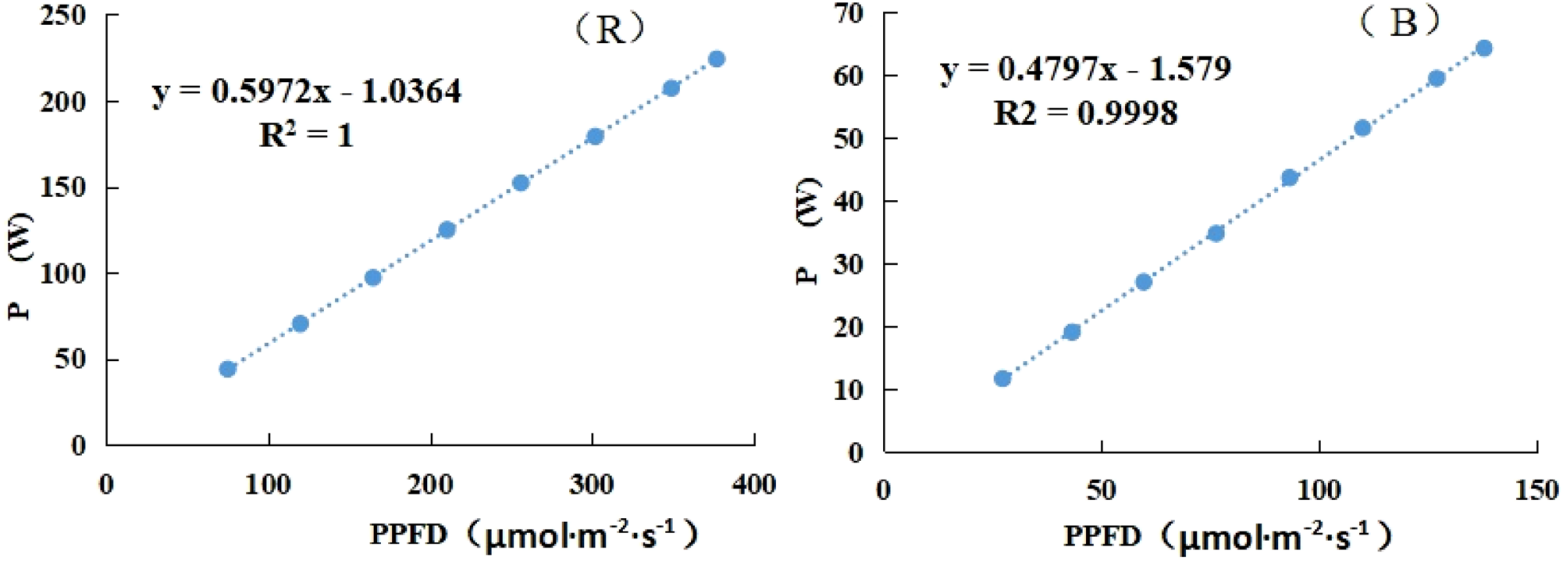
The linear functional relation between light intensity and the actual power (P) of red and blue LEDs.

Germinated seeds of butter leaf lettuce (*Lactuca sativa* L. ‘Flandria’; Rijk Zwaan Co., Netherlands) were sown in sponges and cultivated under 100 μmol·m^−2^·s^−1^ PPFD for 14 days. After that, seedlings were transplanted into hydroponic boxes, supplied with Hoagland’s solution (pH 6.5; EC 1.30 S·m^−1^) with DFT (Deep Flow Technique) and cultivated at 24 °C, relative humidity (RH) 65% and 450 μmol·mol^−1^ CO_2_ in a PFAL. Illumination treatments were conducted using LED panels produced by NERCITA, Beijing, China. The light intensity of R and B could be individually regulated by pulse width modulation (PWM) control. The plants were irradiated with different light spectra provided by R and B (respectively peak at 660 nm and 450 nm as determined by a spectrophotometer; Ocean Optics, model-SD 650, USA.) for 35 days and harvested at 49 days after sowing (DAS), the photoperiod was 16 h per day in all treatments. As shown in Table.1, there were eleven lighting treatments with the same total PPFD of 200 ± 3 μmol·m^−2^·s^−1^ (measured with a light quantum meter at plant canopy level; LI-250A, LI-COR, USA) and different proportions of R and B, respectively recorded as 100%R, 90%R, 80%R, 70%R, 60%R, 50%R, 40%R, 30%R, 20%R, 10%R, and 0%R. The linear functional relation between light intensity and the actual power of LEDs was shown in Fig. 1.

**Table 1:** Light intensity and the proportion of red and blue light in each treatment.

Treatment	Light intensity (μmol·m^−2^·s^−1^)		Electric power consumption (MJ·m^−2^) during the light treatment period (14DAS-49DAS)
	Red light	Blue light	
100%R	200	–	238.70
90%R	180	20	230.78
80%R	160	40	226.04
70%R	140	60	221.31
60%R	120	80	216.57
50%R	100	100	211.83
40%R	80	120	207.09
30%R	60	140	202.36
20%R	40	160	197.62
10%R	20	180	192.88
0%R	–	200	190.23

### Sampling and sample processing

Growth dynamic parameters were measured per week from 21 DAS. Three representative plants were chosen from each treatment at harvest (49 DAS) for plant morphology description^20,21^. Three plants randomly taken from each hydroponic box were regarded as a repetition for index measurements, and there were three repetitions in each treatment. Fresh weight (FW), as well as the contents of chlorophyll (Chl), carotenoid (Car), vitamin C and nitrate were all determined using fresh lettuce samples. Dry weight (DW) and carbohydrate content were determined using the oven-dried lettuce samples (70 °C for 48 h).

### Determination of energy use efficiency

According to Kozai^22^, the EUE (%) and LUE (%) of lettuce planted in the plant factory, and the number of photons required to produce 1 g of dry weight (*p*) as well as the electricity consumed to produce 1 g of dry weight (K) can be determined using the following equations. It was emphasized that the EUE, LUE, *p* and K were all calculated based on the parameters at the harvest time. The initial values before light treatments were recorded as 0.

$$EUE = \frac{{DW \times {W_{{\rm{che}}}} \times S \times D}}{{P \times {\rm{T}}}}\;\;\;\;\;\;\;LUE = \frac{{DW \times {W_{{\rm{che}}}} \times D}}{{{W_{\rm{r}}} \times {\rm{T}}}}$$

$$p = \frac{PPFD \times T}{{DW \times D}},K = \frac{P \times T}{{DW \times D}}$$

***p***—the number of photons required to produce 1 g of weight (μmol·g^−1^), K—the electricity consumed to produce 1 g of weight (J·g^−1^), DW—dry weight (g), W_che_—the chemical energy corresponding to1 gram of dry weight (2 × 10 J.g^−1^ ), S—the cultivation area (m^−2^), D—the planting density (plant·m^−2^), P—the actual power of LED panels (W). T—the cultivation time (s)., W_r_—the photosynthetically active radiation received by plant canopy per unit area (W·m^−2^).

PPFD—photosynthetic photon flux density (μmol·m^−2^·s^−1^).

### Determination of chlorophyll and carotenoid

A total of 0.2 g fresh samples from the mature leaves of lettuce were ground in a mortar and washed using 80% acetone and subsequently filtered (repeated until the leaf turned white). The filtrates were diluted to a total volume of 100 ml with distilled water. The absorbance of the extraction at 470 nm, 645 nm, and 663 nm was respectively measured by a TU-1810s spectrophotometer (PERSEE, Beijing, China). Concentrations of the chlorophyll and carotenoid were determined using the following Eqs. ^23^:

$$Chla\left( {mg/g} \right)\, = \,\tfrac{{(12.72 \times {\rm{OD}}663 - 2.59 \times {\rm{OD}}645){\rm{V}}}}{{1000{\rm{W}}}}$$

$$Chlb\left( {mg/g} \right)\, = \,\tfrac{{(22.88 \times {\rm{OD}}645 - 4.67 \times {\rm{OD}}663){\rm{V}}}}{{1000{\rm{W}}}}$$

$$Car \, \left( {mg/g} \right)\, = \,\tfrac{{((1000 \times {\rm{OD}}4{70} - 3.27 \times {\rm{Chl}}{\rm{.a}} - 104 \times {\rm{Chl}}{\rm{.b}})/229){\rm{V}}}}{{1000{\rm{W}}}}$$

V is the total volume of acetone extract (mL) and W is the fresh weight (g) of the sample.

### Determination of carbohydrate

Sugars: 1.0 g (DW) lettuce shoot sample was extracted in 5 ml 80% (v/v) ethanol for 30 min in a 80 °C water bath and subsequently centrifuged at 12,000** × g** for 10 min (repeated twice). The supernatant was evaporated in a 85 °C waterbath, then the residues were re-dissolved in 20 ml distilled water and subsequently passed through 0.45 μm microporous membrane. Fructose, glucose, and sucrose contents were carried out via the HPLC system (Waters , model-e2695, USA) equipped with 3.5 μm Waters XBridge Amide column (4.6 × 150 mm ) at 30 °C. The mobile phase was acetonitrile/water (75/25, v/v) at a flow rate of 1.0 ml·min^−1^, and the concentrations of the separated sugars were determined according to the corresponding standards (Standard substance center, China)^24^.

Starch: 1.0 g lettuce shoot sample (DW) was mixed with 5 ml 80% (v/v) ethanol and extracted in a 80 °C water bath for 30 min. After centrifuged at 12,000** × g** for 10 min (repeated twice), the precipitate mixed with 3 ml deionized water were boiled for 15 min to gelatinise the starch. After cooling, 2 ml 30% (v/v) HClO_4_ was added and agitated, then the total volume was made to 10 ml by adding distilled water. Afterwards the solution was centrifuged at 12,000** × g** for 10 min (repeated twice) and the supernatant was collected. The glucose liberated in the supernatant was determined with the sulfuric acid anthrone method at a wavelength of 620 nm using TU-1810s spectrophotometer (PERSEE, Beijing, China)^25^.

Crude fiber: 5.0 g lettuce shoot sample (DW) was successively digested with 1.25% sulphuric acid and 1.25% sodium hydroxide, after fully dried, the residue was put in a high-temperature furnace at 550 °C for ashing. Crude fiber was estimated from the loss in weight on ignition of the dried residue using the following equation:

$$Fiber \, \left( \% \right)\, = \,\tfrac{{\rm{loss of weight on ignition}}}{{\rm{weight of sample used}}} \times 10{0}$$

### Determination of nitrate

Nitrate content was measured using the modified method of Cataldo^26^. 0.5 g lettuce shoot sample (FW) mixed with 6 mL deionized water were heated in a 80 °C water bath for 30 min, after cooling and filtered twice, the filtrate was collected and supplemented with deionized water to make total volume of 100 mL. Afterwards, 0.1 mL solution was taken and mixed with 0.4 mL of 5% (w/v) salicylic acid (in pure H_2_SO_4_) and 9.5 mL of 8% NaOH, finally the nitrate content was measured with TU-1810s spectrophotometer (PERSEE, Beijing, China) at a wavelength of 410 nm.

### Determination of vitamin C

Vitamin C content was measured using the modified method of Gahler^27^. 0.2 g lettuce shoot samples (FW) was mixed with 15 mL of 4.5% aqueous phosphoric acid and shaken at 300 rpm for 30 min in the darkness and then centrifuged at 16,000 *g* for 10 min. The supernatants was eluted with 0.21% phosphoric acid at a flow rate of 0.8 mL/min, and the concentration of vitamin C was determined via the HPLC system (Agilent, model-1100, USA) equipped with C18 column (inner diameter 4.6 mm, length 250 mm, particle diameter 5 μm, Restek USA, Bellefonte, PA, USA) at 254 nm against ascorbic acid standards (Standard substance center, China).

### Statistical analysis

Statistical analysis was performed using SPSS 11.0 software (SPSS Inc., Chicago, USA). Significance at the 0.05 significance level were conducted by Tukey’s multiple range test.

## Results and analysis

### Morphology and growth dynamics

**Figure 2:**
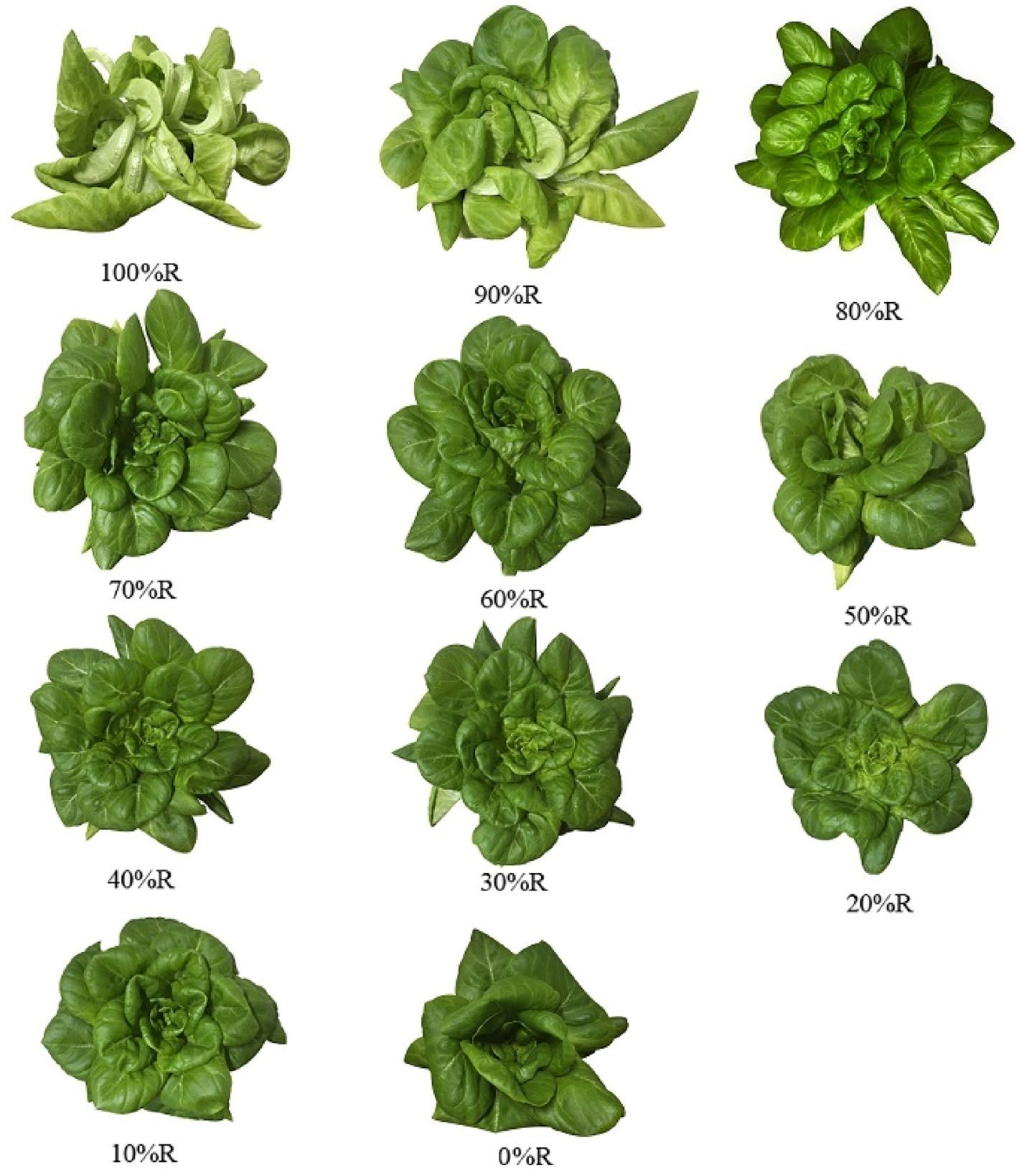
Morphology of lettuce under different light treatments at harvest (49DAS). (The URL link of the software used in the figure is http://adobe.com/cn/products/photoshop.html).

As shown in Fig. 2, lettuce morphology differed obviously with the varied R and B proportions. When the proportion of R was less than 70%, no obvious petiole distortion was observed. On the contrary, palpable petiole distortion appeared when R proportion was more than 70% and the distortion was aggravated with the increase of R proportion. The minimum leaf number of lettuce was observed under pure B treatment, while the maximum leaf number was detected in lettuce under 70%R, 80%R and 90%R treatments (no significant difference existed among the three treatments). Roughly speaking, the color of leaves became lighter and the number of leaves increased with the increase of R proportion.

**Figure 3:**
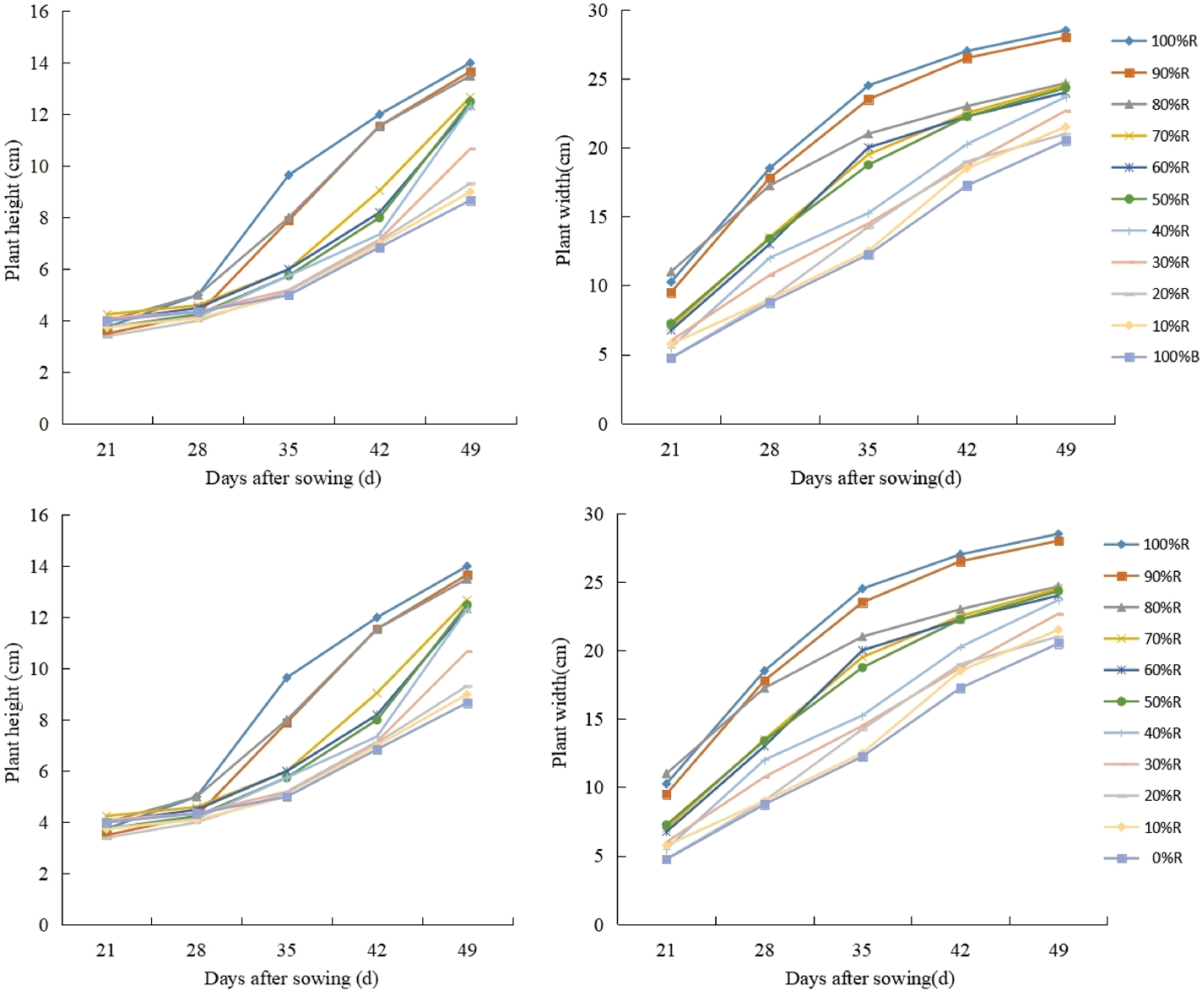
Plant height and plant width under different light treatments at harvest (49DAS).

As shown in Fig. 3, the highest average growth rate of plant height during the whole treating period (from 21 to 49 DAS) was observed in lettuce cultivated with 100%R treatment, which was 0.5 cm·d^−1^, 61.5% higher than that under 0%R treatment. In 80% R, 90% R and 100%R treatments, the highest elongation rate of lettuce plants appeared at the stage from 28 DAS to35 DAS, and the highest average elongation rate was 0.66 cm·d^−1^. In contrast, for the other treatments, the highest elongation rate of plants was detected at the stage from 35 to 49 DAS, and the highest average elongation rate was 0.71 cm·d^−1^. As regards of the growth dynamic of plant width (Fig. 3), it was found that the growth trend of plant width basically appeared the same among all the treatments, and the highest average lateral expansion rate (1.02 cm·d^−1^) was detected in plants during the stage from 21 to 35 DAS.

### Chlorophyll and carotenoid contents

**Figure 4:**
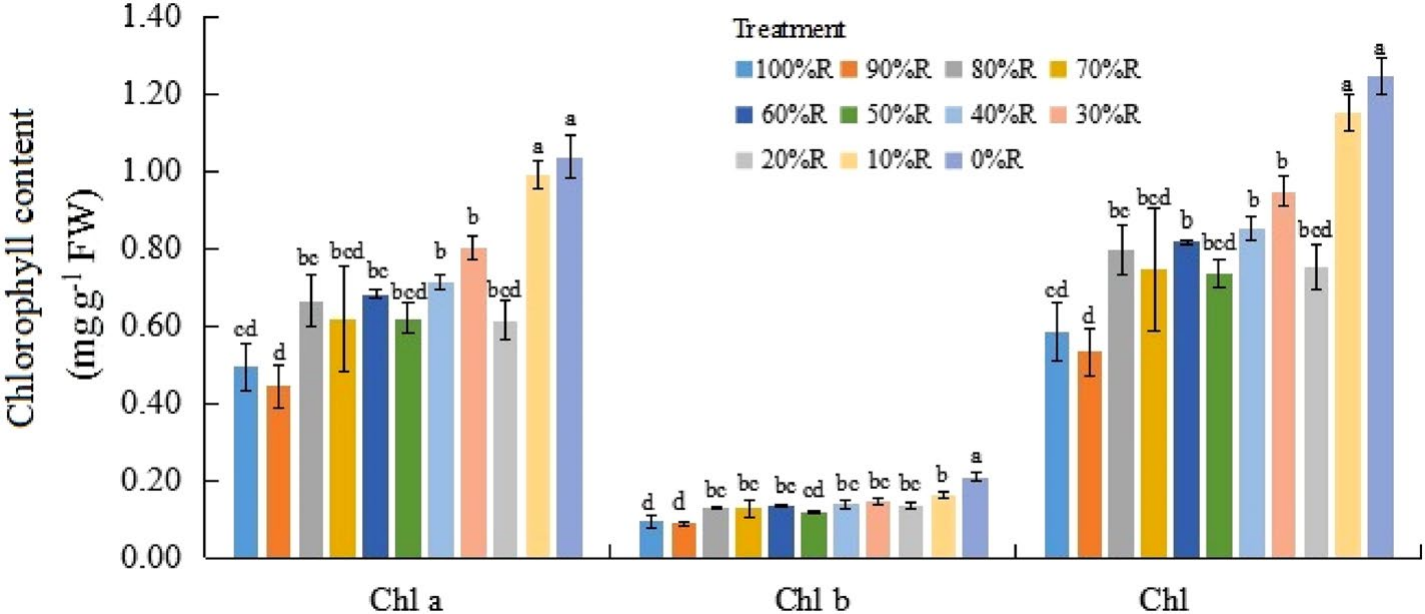
Chlorophyll (Chl) contents of plants grown under different light treatments (at harvest). Different letters for the same parameter indicate significant differences at the 5% level, according to the Tukey’s test (n = 3). The bars represent the standard errors.

**Figure 5:**
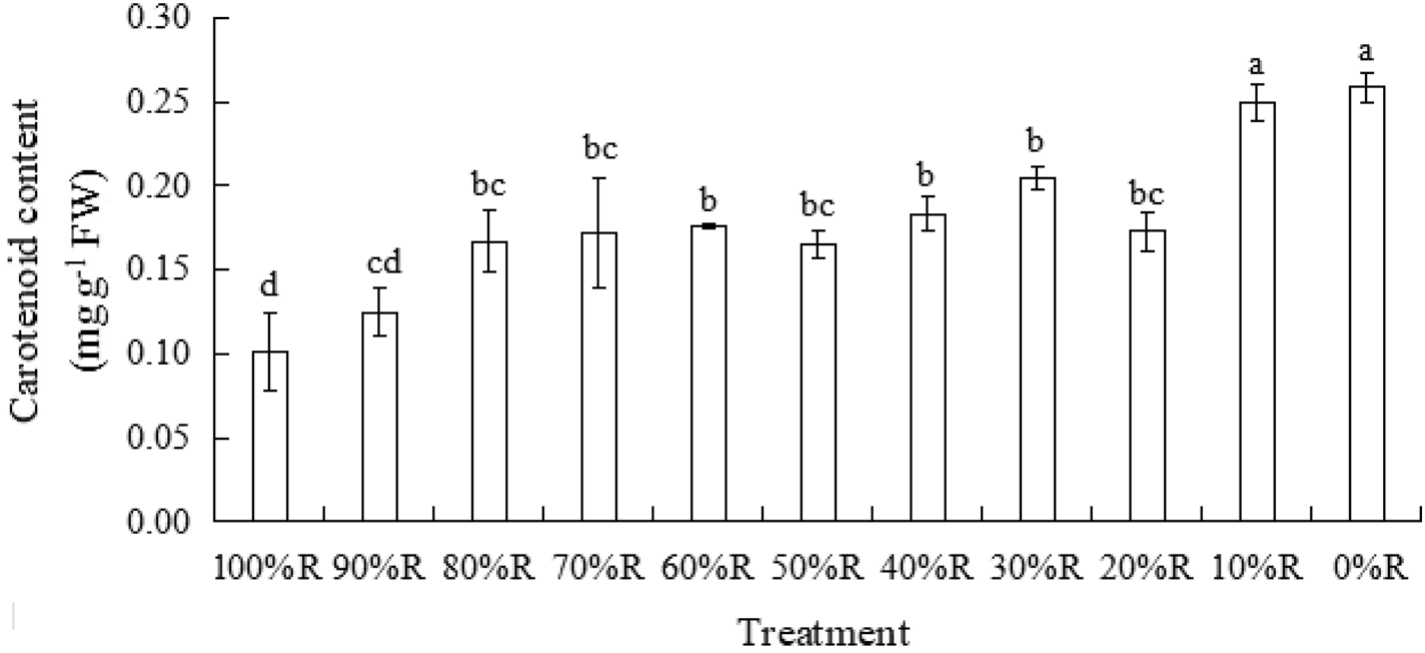
Carotenoid (Car) content of plants grown under different light treatments (at harvest). Different letters for the same parameter indicate significant differences at the 5% level, according to the Tukey’s test (n = 3). The bars represent the standard errors.

The trend presented in Figs. 4 and 5 roughly showed that the contents of Chl a, Chl b and Car in lettuce leaves increased with the decrease of R proportion. Among all the treatments, the highest contents of Chl and Car were both detected in lettuce leaves treated with 0%R. In contrast, Chl and Car contents in lettuce leaves under 100%R appeared the minimum values (or not significantly different with the minimum value). It indicated that B played an important role in chlorophyll and carotenoid synthesis.

### Energy use efficiency

**Figure 6:**
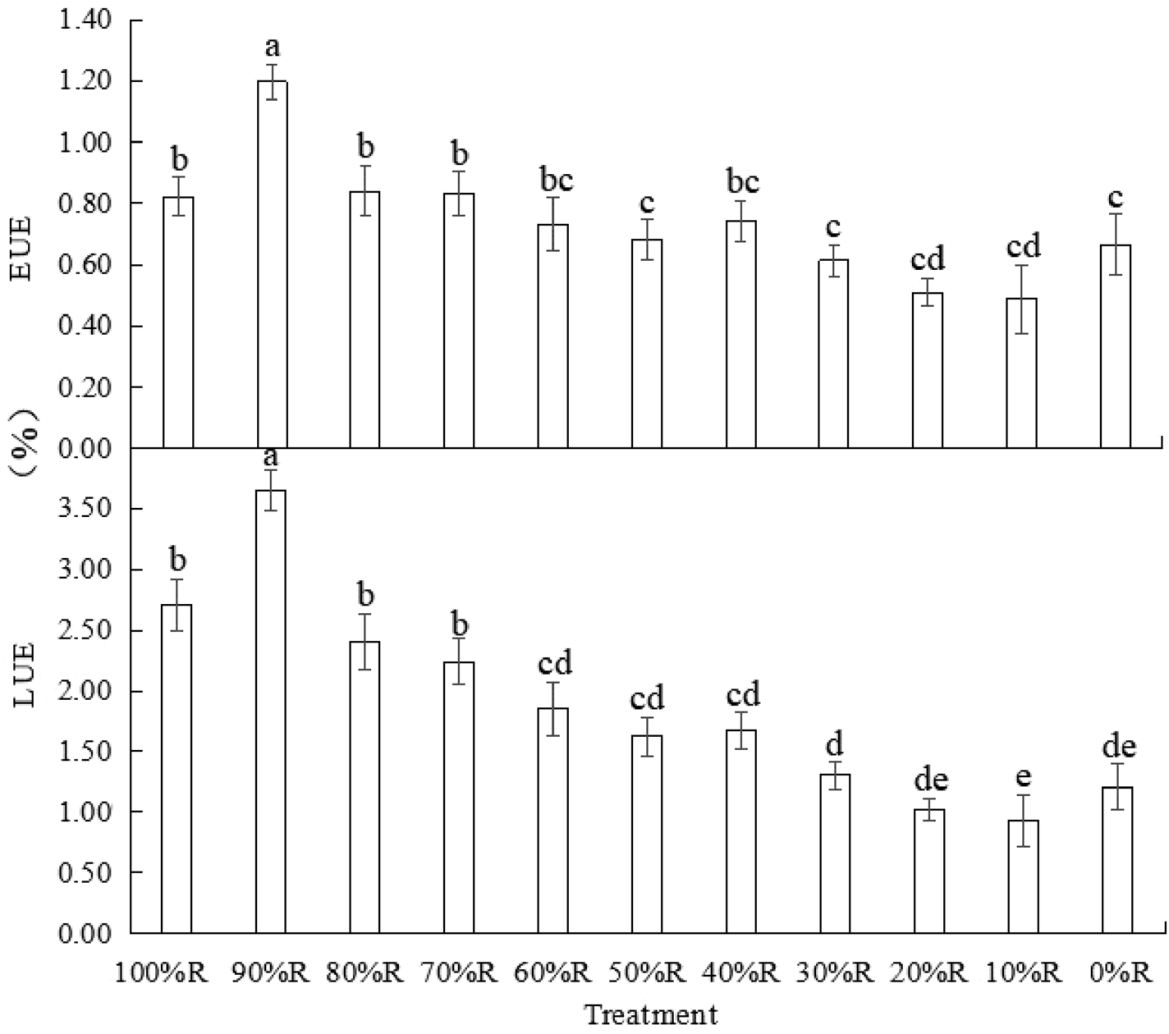
LUE and EUE of plants grown under different light treatments (at harvest). Different letters for the same parameter indicate significant differences at the 5% level, according to the Tukey’s test (n = 3). The bars represent the standard errors.

As shown in Fig. 6, except 100%R and 0%R, LUE and EUE of lettuce were roughly enhanced as the proportion of R increased. The highest LUE and EUE were both detected in lettuce under 90% R treatment, which were respectively 3.64% and 1.20%. Compared with pure B, adding R helped to enhance LUE and EUE, but LUE and EUE in lettuce under 100%R was significantly lower than the maximum value observed in lettuce treated with 90%R. As shown in Table 2, the least number of photons required to produce 1 g of dry weight was 2.92 mol, and the least electricity amount consumed to produce 1 g of dry weight was 1.67 MJ. The minimum values of *p* and K were both detected in lettuce treated with 90%R, while the maximum values of them were both observed in lettuce treated with 10%R, and the maximum values were 2.4 ~ 3.1 times the minimum values.

**Table 2:** Photons and electric power consumption for producing per unit weight of lettuce harvested at 49 DAS. *p*: photons consumed for producing per gram of lettuce in a unit of planting area; k:electric energy consumed for producing per gram of lettuce in a unit of planting area.

Treatment	***p***(mol·g^−1^)		K(MJ·g^−1^)	
	Dry weight	Fresh weight	Dry weight	Fresh weight
100%R	4.11	0.14	2.44	0.09
90%R	2.92	0.09	1.67	0.05
80%R	4.24	0.12	2.38	0.07
70%R	4.37	0.14	2.40	0.08
60%R	5.09	0.18	2.74	0.09
50%R	5.60	0.17	2.94	0.09
40%R	5.23	0.16	2.69	0.08
30%R	6.49	0.20	3.26	0.10
20%R	8.01	0.28	3.93	0.14
10%R	8.57	0.28	4.10	0.14
0%R	6.39	0.25	3.02	0.12

### Hexose and sucrose contents

As shown in Table 3, the contents of glucose and fructose in leaves under pure R were not significantly different from that under pure B, but the sucrose content in leaves under pure R was significantly higher than that under pure B. With the increase of R proportion, the contents of glucose and fructose in lettuce leaves decreased first and then increased. The contents of glucose and fructose reached the highest level under 60%R, 70%R, 80%R and 90%R treatments, and there was no significant difference among the four treatments. The maximum contents of glucose and fructose respectively increased by 71.76% and 187.97% compared with the minimum contents of them. As regards of sucrose, there was no significant difference among treatments with R proportion greater than 50%, so was those treatments with R proportion less than 50% (50%R treatment included). The sucrose content in lettuce under mixed RB with more than 50%R was significantly higher than that under mixed RB with less than 50% R (50%R included). It indicated that R proportion exceeding 50% in mixed RB was beneficial to the accumulation of hexose and sucrose in lettuce.

**Table 3:** The contents of glucose, fructose and sucrose in lettuce at harvest (49DAS). Values for the same parameter with different letters significantly differ at the 5% level (by Tukey’s test, n = 3).

Treatment	Glucose (g·kg^−1^ DW)	Fructose (g·kg^−1^ DW)	Sucrose (g·kg^−1^ DW)
100%R	24.45 b	38.71 c	24.54 a
90%R	29.02 ab	49.07 a	27.39 a
80%R	32.64 a	49.40 a	25.74 a
70%R	23.66 b	45.40 ab	22.41 a
60%R	29.11 ab	50.15 a	23.69 a
50%R	19.24 bc	40.22 b	18.12 b
40%R	21.05 b	41.71 b	16.04 b
30%R	19.70 bc	28.50 d	17.29 b
20%R	19.01 bc	26.68 d	18.58 b
10%R	18.80 c	36.13 c	17.64 b
0%R	23.00 b	37.90 c	17.14 b

### Carbohydrate contents

**Figure 7:**
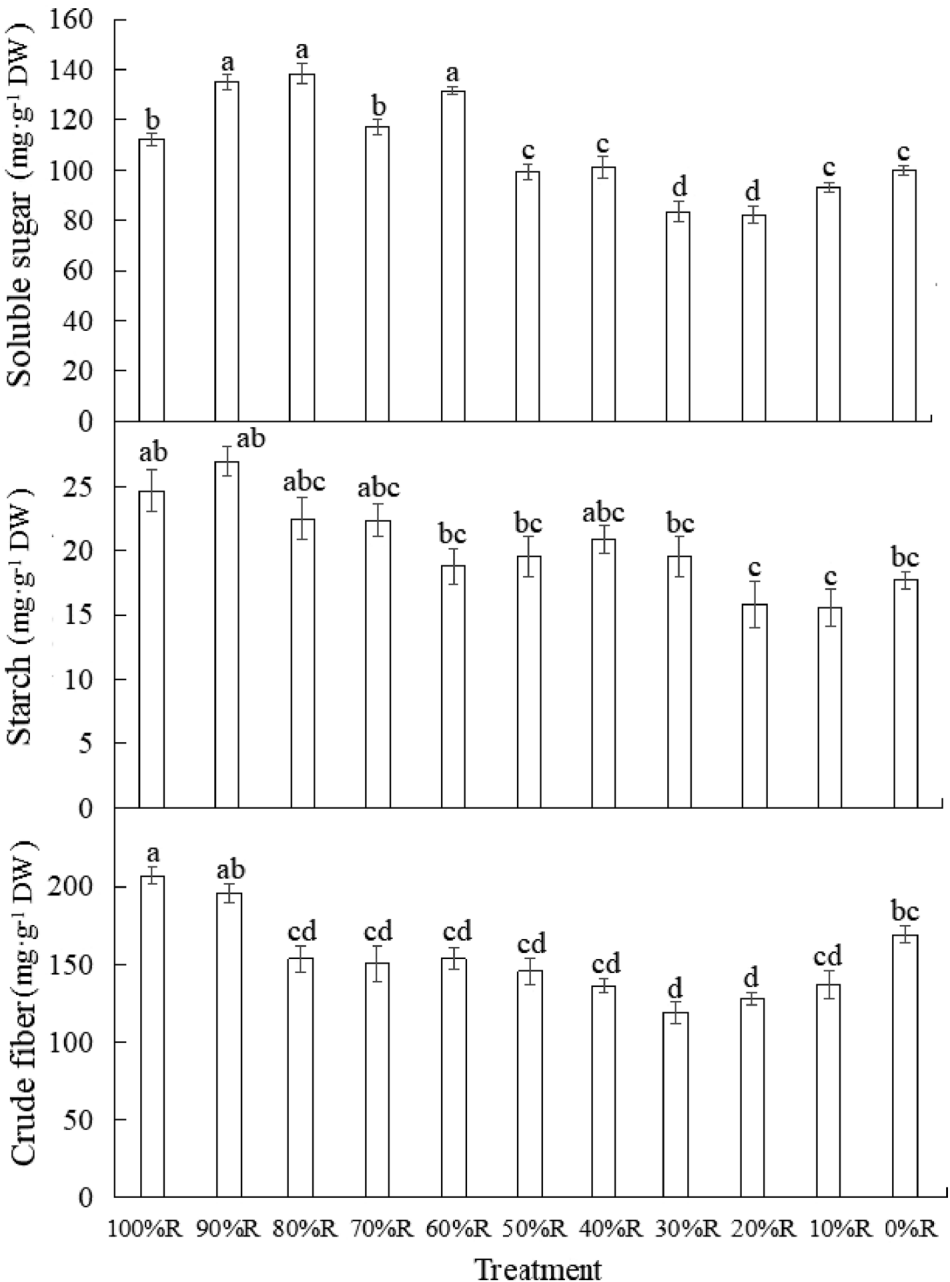
The contents of soluble sugar, starch and crude fiber of lettuce plants grown under different light treatments (at harvest). Different letters for the same parameter indicate significant differences at the 5% level, according to the Tukey’s test (n = 3). The bars represent the standard errors.

As shown in Fig. 7, soluble sugar content in lettuce under pure R was significantly higher than that under pure B. The highest content of soluble sugar was observed in lettuce treated with 60%R, 80%R and 90%R, no significant difference existed among the three treatments. The soluble sugar content of lettuce in the treatments from 10%R to 50%R is lower than or close to that in 0%R treatment, indicating that the proportion of B exceeding 50% in mixed RB was not conducive to the accumulation of soluble sugar in lettuce. Starch is one of the direct products of photosynthesis. The starch content showed an overall upward trend with the increase of the proportion of R, but did not reach a significant level. The highest starch content appeared in lettuce treated with 90%R and 100%R, and the lowest starch content was detected in plants under 10%R and 20%R treatments, the maximum value increased by about 25% compared with the minimum value. With the proportion of R increased, the crude fiber content of lettuce decreased first and then increased. The highest value appeared in plants treated with 90%R and 100%R. No significant difference existed among the treatments from 10%R to 80%R.

It is generally believed that the higher soluble sugar content and the lower crude fiber content in lettuce leaves resulted in better taste of lettuce (Fillion and Kilcast, 2002). Lettuce treated with 60%R and 80%R possessed the highest soluble sugar content as well as the lowest crude fiber content, thus it was speculated that lettuce under 60% R and 80% R treatments tasted the most sweet and crisp.

### Vitamin C content

**Figure 8:**
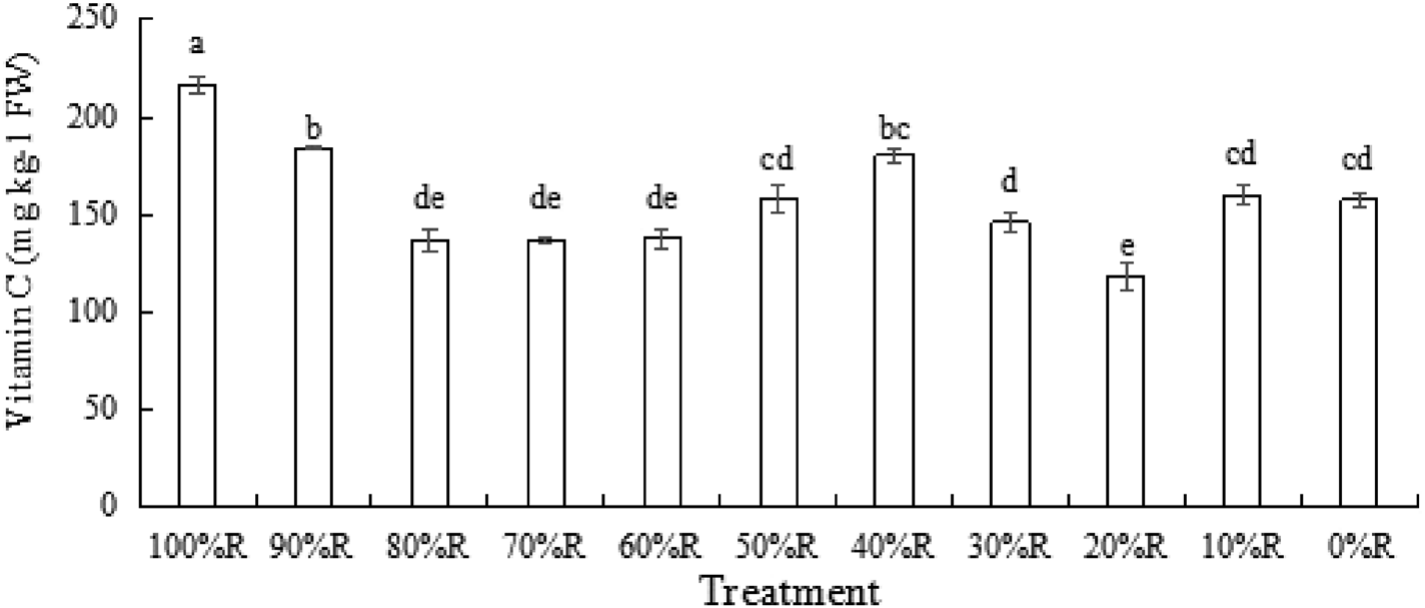
The content of Vitamin C of lettuce plants grown under different light treatments (at harvest). Different letters for the same parameter indicate significant differences at the 5% level, according to the Tukey’s test (n = 3). The bars represent the standard errors.

As shown in Fig. 8, vitamin C content in lettuce fluctuated with the varied R or B proportion, and no obvious trend was detected in vitamin C content with the increased proportion of R. It might imply that vitamin C was sensitive to light spectrum. The highest vitamin C content appeared in lettuce treated with 100%R, significantly higher than that in plants under the other treatments in the study, indicating that pure R promoted the synthesis or accumulation of vitamin C in lettuce.

### Nitrate contents

**Figure 9:**
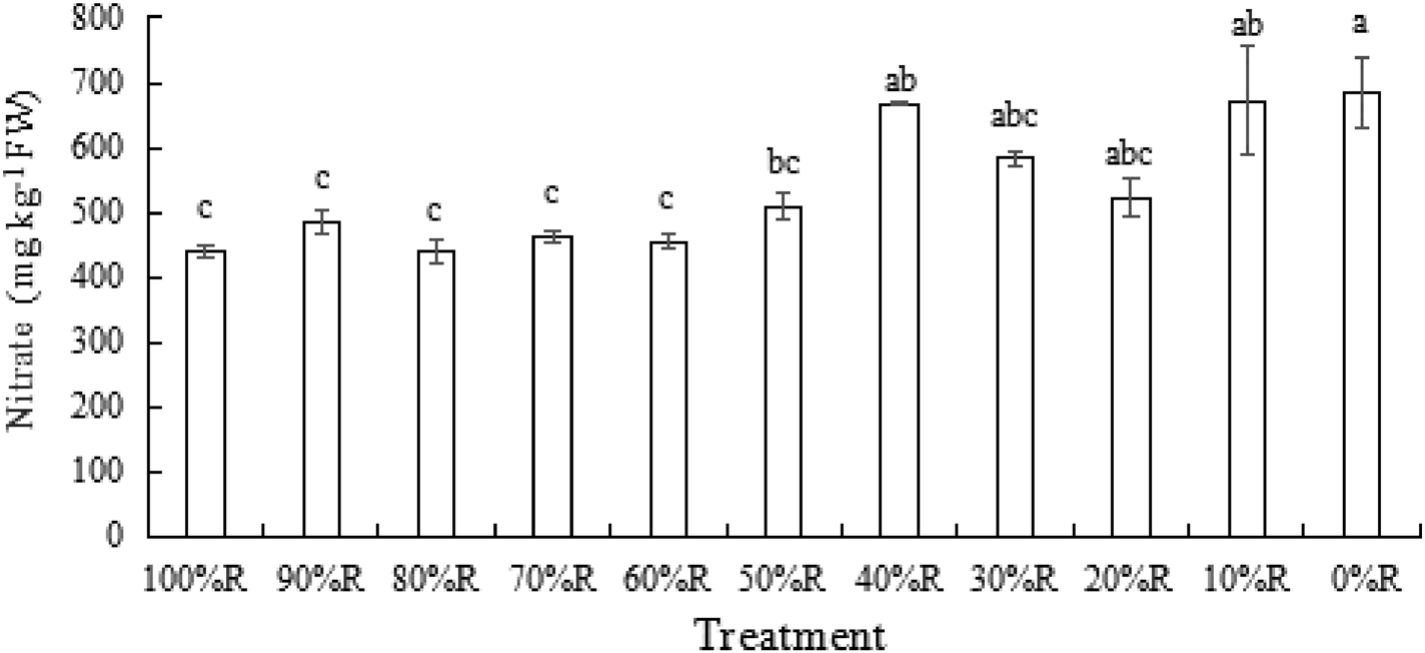
The contents of nitrate of lettuce plants grown under different light treatments (at harvest). Different letters for the same parameter indicate significant differences at the 5% level, according to the Tukey’s test (n = 3). The bars represent the standard errors.

As shown in Fig. 9, lettuce under 0%R displayed the highest nitrate content (684.79 mg·kg^−1^), followed by 10%R. The lowest nitrate content (439.36 mg·kg^−1^) appeared in lettuce treated with 80%R. However, no significant difference was observed among the treatments from 40%R to 0%R. It might indicate that the proportion of B exceeding 50% in mixed RB was not conducive to the decomposition of nitrate in lettuce.

## Discussion

In the present study, obvious differences were detected in the presence and absence of B for the plant shape index (plant height/plant width) and plant morphology. The plant shape index under the 100%R treatment was higher compared with those treatments containing B throughout the whole treating period. Hernández^16^ conducted six RB treatments that were 0B:100R%, 10B:90R%, 30B:70R%, 50B:50R%, 75B:25R%, 100B:0R%, declaring that the hypocotyl length of cucumber seedlings decreased with the increase of B fraction. Similar results have been detected in other previous studies^28–31^. It proved that B inhibited leaf expansion while R induced lettuce leaf elongation, reducing B could increase the elongation of stem and leaves. However, it is worth noting that R alone resulted in abnormal leaf shape with petiole distortion, which has also been raised in the study of Son and Oh^15^.

The energy consumption of artificial light source is an important factor restricting the development of closed plant factory, where R and B LEDs haven been used as main light qualities. As regards of irradiation strategy in a plant factory, numerous studies on lighting strategies including light intensity, light period and light quality have been reported, however, the LUE or EUE of plants have rarely been concerned. Since R and B LED light has different quantum efficiency and photoelectric conversion efficiency, combinations of R and B with the same total PPFD but different proportions of R and B show varied energy consumption. In order to improve the energy efficiency of the system, it is necessary to study the effects of different R and B proportions on not only the quality but also the EUE and LUE of plants. The results in the current study showed that the LUE and EUE of lettuce were enhanced when R was included, but LUE and EUE in lettuce under 100%R was significantly lower than the maximum value observed in lettuce treated with 90%R. It might indicate that R could increase the energy use efficiency of lettuce, but the potential of R to improve the energy use efficiency seemed higher when 10%B participated. It was of interest to see that compared with pure B, a small amount of supplemented R (i.e., the 10%R and 20%R treatments) reduced the energy utilization efficiency and increased the photon and electricity required for the production of per unit weight of lettuce. With the continuous increase of R proportion, the energy utilization efficiency began to rise. The result further suggested that the relationship between R and B when acting on plants was not independent but interactive. Similar results have been referred in previous studies. For example, Dougher and Bugbee^14^ reported that adding only 2% B could effectively promote biomass accumulation in lettuce treated with mixture RB. Trouwborst^32^ reported that abnormal photosynthetic functioning in cucumber plants resulted by monochromatic R could be reversed by adding a small fraction of B.

The absence of B was detrimental to chlorophyll biosynthesis in many plant species such as cucumber, spinach and wheat etc.^33,34^. Lots of previous studies confirmed that B promoted the biosynthesis of chlorophyll and played a major role in the generation and move of chlorophyll in plant leaves^35–39^. Some researchers considered that B had a qualitative effect, rather than a quantitative effect, on the chlorophyll biosynthesis of plants^29,40^, since the chlorophyll content did not sustaintly increase with the B proportion increased in those findings. The results in the current study was opposite with the findings mentioned above, whereby the chlorophyll content gradually increased with increasing B proportion until 0%R (100%B). Son and Oh^15^ reported that the SPAD value of ‘Sunmang’lettuce gradually increased with increasing B ratios, which supported the result in the current study. It may indicate that chlorophyll content of different cultivars respond differently to light quality. Additionally, it was observed in the current study that not only the contents of Chl, but also Car in lettuce leaves increased with the increase of B proportion, and the color of leaves became greener with the increase of B proportion. Finally, abundant photosynthetic pigments produced by monochromatic B was not positively correlated with LUE of lettuce, indicating that R wavelength was required for transferring and transforming the light energy during the whole photosynthetic process.

Ohashi-Kaneko^4^ reported that vitamin C accumulation in lettuce was increased by supplemental B. Inconsistent results observed in the present study demonstrated that 100%R caused significantly higher vitamin C content compared with the other treatments, indicating that pure R irradiation in the absence of B was effective in stimulating the synthesis of vitamin C in lettuce. However, we also found that when plants were subjected to combined RB with B fraction of 30%-60%, the content of vitamin C did increase with the increase of B fraction. Thus, we concluded that the inconsistency with the previous study might be due to the limitation of experiment treatments. It implied that the response of vitamin C in lettuce was sensitive to the proportions of R and B.

As a harmful substance for human health, the accumulation of nitrate in plants is affected by plant genotype, and the supply of light, water and nitrogen. The current maximum limit standard of EU on food pollution stipulates that the maximum allowable nitrate content of lettuce harvested from October 1 to March 1 is 4000 mg kg^−1^, while those harvested from April 1 to September 31 is 2500 mg kg^−1^^41,42^. Supplemental R has been reported to reduce the nitrate content in red leaf lettuce^4^. Similar result was observed in our study, lettuce irradiated with 0%R showed the highest nitrate (684.79 mg kg^−1^) content followed by 10%R (671.45 mg kg^−1^) and the lowest nitrate content was detected under 80%R treatment (439.36 mg kg^−1^). Of course, in terms of absolute nitrate content, further reduction by regulating other cultivation conditions is possible. R may reduce nitrate content in lettuce by activating the nitrate reductase via receptors such as phytochromes^43^. However, no significant difference was detected in nitrate content when R fraction increased from 0 to 40%. It might indicate that the nitrate decomposition could be promoted more effectively when more than 50%R was added.

In addition to nitrate decomposition, we also found that R proportion exceeding 50% in mixed RB was beneficial to the accumulation of hexose and sucrose in lettuce. Hogewoning^9^ also found that increasing B fraction within a certain range (0–50%) could increase photosynthesis capacity in cucumber seedlings. Therefore, it may be concluded that “50%R” or “50%B” in mixed RB is a breakthrough point for the trend of some physiological and nutritional indexes.

## Conclusion

The proportions of R and B in mixed RB affected lettuce cultivation in the ways of energy use efficiency, plant morphology, and nutrient substance. Compared with B, lettuce had higher energy utilization rate under R, however supplemental 10%B enhanced the total energy use efficiency of lettuce, and 90%R was the optimal treatment in terms of energy use efficiency. The palpable petiole distortion appeared in lettuce when R proportion was more than 70% and the distortion was aggravated with the increase of R proportion. Obvious decline of sucrose content and rise of nitrate content were observed in lettuce when R proportion was less than 50%. 80%R was the recommended treatment as far as lettuce qualities were concerned.

## Data Availability

The data used to support the findings of this study are included within the article. The study complies with local and national regulations. No collection of seeds or plants are involved in this study. The study complies with local and national regulations. No collection of seeds or plants are involved in this study.
